# AI Tools for Teaching the Safe Administration of Medications in Nursing: A Scoping Review

**DOI:** 10.3390/nursrep16040146

**Published:** 2026-04-21

**Authors:** Wínola Dafny Douglas de Oliveira, Maria Eduarda Leite Pinto Ghirotti, Álvaro Francisco Lopes de Sousa, Adaiele Lúcia Nogueira Vieira da Silva, Herica Emília Félix de Carvalho, Marília Duarte Valim, Aires Garcia dos Santos Júnior

**Affiliations:** 1 Câmpus de Três Lagoas, Universidade Federal de Mato Grosso do Sul, Três Lagoas 79070-900, MS, Brazil; winola.dafny@ufms.br (W.D.D.d.O.); maria_leite@ufms.br (M.E.L.P.G.); aires.junior@ufms.br (A.G.d.S.J.); 2 Public Health Research Centre, Comprehensive Health Research Center, CHRC, REAL, NOVA University Lisbon, 1050-091 Lisbon, Portugal; 3 Câmpus de Campo Grande, Universidade Federal do Mato Grosso do Sul, Campo Grande 79070-900, MS, Brazil; adaiele.silva@gmail.com; 4 School of Nursing, Federal University of São Carlos (UFSCar), São Carlos 13565-905, SP, Brazil; marilia.duarte.valim@gmail.com; 5 Nursing Program, Universidade Estadual do Maranhão (UEMA), Coroatá 65400-000, MA, Brazil; hericacarvalho@professor.uema.br

**Keywords:** artificial intelligence, nursing, patient safety, nursing education

## Abstract

**Background**: Safe medication administration is a fundamental aspect of nursing practice and a core component of patient safety. However, systemic failures, workload pressures, and educational gaps continue to contribute to medication errors, posing persistent challenges for healthcare systems. In this context, innovative educational technologies, particularly Artificial Intelligence (AI), have emerged as promising strategies to support the development of competencies related to safe medication administration. **Methods**: This scoping review aimed to map evidence on AI-based tools used to teach safe medication administration in nursing. The review was conducted in accordance with the Joanna Briggs Institute (JBI) methodology and reported following the PRISMA-ScR guidelines. Searches were performed in PubMed, Scopus, Web of Science, LILACS, and Google Scholar, covering studies published between 2010 and October 2025 in English, Portuguese, and Spanish. Study selection was conducted in two stages, followed by standardized data extraction. **Results**: A total of 545 records were identified, of which only two studies met the eligibility criteria. The included studies, conducted in Israel and South Korea, evaluated a microlearning chatbot and Large Language Model (LLM)-based tools designed to support teaching safe medication administration. Both studies demonstrated improvements in knowledge and performance in tasks and simulations related to the medication process, as well as positive acceptability among participants. However, neither study assessed direct clinical outcomes, such as reductions in medication errors or preventable adverse events. **Conclusions**: Although AI-based educational tools show potential to enhance competencies related to medication safety in nursing, the available evidence remains limited. Further robust, multicenter, and comparative studies are needed to evaluate their impact on clinical outcomes and to support their integration into nursing education and practice.

## 1. Introduction

Safe medication administration is a foundational competency of nursing practice and a pillar of patient safety [1]. However, this process is inherently complex and high risk, taking place in a dynamic clinical environment marked by interruptions and competing demands that impose a high cognitive load on professionals [1]. In this context, medication errors persist as a critical failure within global health systems [2,3]. It is estimated that one in twenty patients experience harm related to such errors, and one quarter of these events result in serious or fatal consequences [2,3,4]. These incidents not only cause preventable harm but also impose substantial costs on health systems, constituting a chronic, high-priority challenge [2,3,4].

Patient safety, according to the World Health Organization, involves reducing the risk of unnecessary harm to an acceptable minimum and is considered inseparable from the quality of care [2,5]. In this sense, medication administration, often under the nurse’s responsibility, ranks among the processes most susceptible to failure and represents a strategic lever for consolidating a culture of safety in healthcare services [6]. Positioned on the front lines of care, nurses act as the last line of defense between error and harm, reinforcing the need for continuous training, technological support, and systems that enable safe decision-making [6].

Advancing patient safety, however, goes beyond the mere prevention of errors. It requires a systems-based approach aimed at creating processes, cultures, and work environments that actively reduce the likelihood of harm [7]. In the context of medication administration, this requires nursing professionals to develop higher-order competencies, such as critical thinking to question ambiguous prescriptions, situational awareness to identify imminent risks, and effective communication skills to interact with a multidisciplinary team [7]. A culture of safety depends less on human perfection and more on professionals’ capacity to anticipate, recognize, and mitigate the risks inherent in the medication process [1], aligning with global challenges to reduce medication-related harm.

Despite decades of pedagogical emphasis on the “rights” of medication administration, the persistence of errors suggests that traditional teaching models, while essential, have reached a ceiling of effectiveness [7,8]. The fundamental gap lies in the difficulty of transferring theoretical knowledge acquired in controlled environments into consistent and safe application within the complexity of clinical practice [8]. Real-world challenges, such as polypharmacy in older adults, dose calculations for high-alert medications, and decision-making under pressure, require more than memorizing protocols. They point to an urgent need for pedagogical innovations that transcend the conventional classroom and infrequent, low-fidelity simulation labs [8].

As the third International Patient Safety Goal, preventing medication errors is a primary concern in healthcare services because it is directly related to the occurrence of preventable adverse events [2,5]. Among the most frequent contributing factors are the absence of well-defined institutional protocols and communication failures among members of the multiprofessional team, which compromise the continuity and safety of care [6]. Consequently, it is essential to implement preventive measures, standardize clear protocols, and strengthen continuing education to ensure that professionals remain up to date, competent, and committed to safe practices in the medication administration process [9].

In this scenario, Artificial Intelligence (AI) is emerging as a catalyst for a new approach to nursing education [10,11]. AI’s potential lies in its ability to create personalized, adaptive, and safe learning environments that mirror clinical challenges [10,11]. AI can provide automated, real-time feedback, generate realistic simulations, and, crucially, support clinical reasoning through language models and intelligent tutoring systems [10]. Tools such as instructional chatbots based on microlearning enable deliberate practice by offering focused repetition with immediate feedback, something logistically unfeasible for human educators in large cohorts [11]. Complementarily, large language models (LLMs), such as ChatGPT, function as conversational tutors that can enhance clinical reasoning and support the review of critical steps in the medication process, provided they are used under faculty oversight [12,13].

Nevertheless, despite this promising potential, translating theory into evidence-based practice remains the primary current barrier. The critical gap in the literature is not only a scarcity of studies but also the absence of research demonstrating a causal connection between AI-based educational interventions and improvements in measurable clinical outcomes [8,9,10,11,12,13,14]. The current literature, though optimistic, lacks robust studies evaluating the translation of knowledge gains into an actual reduction in medication errors or near misses at the bedside [8,9,10,11,12,13,14]. Without a critical synthesis of the existing evidence, there is a risk of adopting technologies without understanding their true mechanisms of effectiveness, limitations, and real impact on patient safety. For nursing educators, a paradigm shift is underway, beginning with a broader understanding of how generative AI can be applied in academic settings, as well as how to address its potentially disruptive systemic impacts [15].

In light of this, this scoping review aims to map the evidence on the use of artificial intelligence (AI) in teaching the safe administration of medications in nursing, with a focus on patient safety.

## 2. Materials and Methods

A scoping review was conducted to map the extent, range, and nature of the evidence on the use of artificial intelligence in teaching the safe administration of medications in nursing. The review was developed in accordance with the methodological guidance of the Joanna Briggs Institute (JBI) and is reported following the Preferred Reporting Items for Systematic Reviews and Meta-Analyses extension for Scoping Reviews (PRISMA-ScR), explicitly addressing the review objectives, eligibility criteria, information sources, study selection, and data charting processes. The review protocol was defined a priori, including the research question, eligibility criteria, information sources, search strategy, selection procedures, data charting methods, and synthesis approach, and was prospectively registered on the Open Science Framework (OSF) (https://osf.io/g9c5a accessed on 2 October 2025). Study selection was performed in two sequential stages by two independent reviewers: initial screening of titles and abstracts to identify potentially relevant records, followed by full-text assessment for final inclusion.

The research question was structured using the PIO framework: -Population: nursing students and professionals; -Intervention: AI-based tools applied to teaching or training for the safe administration of medications in nursing (including symbolic AI, machine learning, deep learning, chatbots, LLMs/generative AI, intelligent tutoring systems, and microlearning with intelligent components, among others); -Outcomes: educational (knowledge, skills, performance in tasks or simulations, retention) and implementation-related (acceptability, usability, feasibility), and, when available, patient safety outcomes (for example, medication errors, near misses, adherence to the “rights” of medication administration).

Eligible studies included original research (quasi-experimental, experimental, observational, pilot or feasibility, or mixed methods) or implementation reports with a systematic evaluation of educational effects involving nurses and/or nursing students. Mixed samples were accepted when results specific to nursing were clearly presented or could be extracted. Interventions based on AI with an educational purpose directly related to medication administration (for example, dose calculation, checks, preparation or infusion, drug and route identification, safe documentation) and explicitly focused on patient safety were included.

Publications from 2010 onward in Portuguese, English, or Spanish with full-text access were considered. This language restriction was applied to ensure accurate data charting and interpretation by the review team, whose members are proficient in these languages [16,17]. In addition, resource constraints and the absence of professional translation support limited the feasibility of systematically reviewing studies published in other languages. Similar exclusion criteria have been adopted in previous scoping reviews in emerging fields [16,17]. Exclusion criteria comprised studies using AI solely for clinical or care purposes (for example, decision-support systems, computerized prescribers, pharmaceutical validation) without an educational component; studies outside the nursing field when results could not be separated; opinion pieces, editorials, letters without data, guidelines without evaluation of educational interventions, and secondary reviews; as well as research in which the teaching content was not related to safe medication administration.

Information sources included PubMed/MEDLINE, Scopus, Web of Science Core Collection, LILACS, and Google Scholar (to expand coverage by screening the first 100 results sorted by relevance, corresponding to the first 10 pages), as well as manual searches of the reference lists of included studies. Searches were executed and last updated on 2 October 2025. The search strategy combined controlled descriptors (MeSH/DeCS) and free-text terms using Boolean operators, truncation, and field-specific queries (title, abstract, subject), without restrictions on study design, applying filters for publication year ≥ 2010 and languages English, Portuguese, and Spanish. Detailed search strategies for each database were recorded and archived, and the full search strategies for all databases are provided in Table A1. The search strategy was developed and refined in collaboration with an experienced health sciences librarian or information specialist to optimize sensitivity and specificity and strengthen the Boolean combinations.

Records were exported in RIS or BibTeX formats to a reference manager (Zotero or EndNote (Corporation for Digital Scholarship, Vienna, VA, USA; https://www.zotero.org; accessed on 1 October 2025)), and duplicate removal was performed in two steps: automated detection (title, author, year, DOI) followed by manual verification (title, first author, journal, DOI). Study selection was conducted in two stages by two independent reviewers: title and abstract screening according to the eligibility criteria, followed by full-text review of potentially relevant records. Discrepancies were resolved by consensus through discussion between the two reviewers, with the involvement of a third reviewer when necessary. Study selection and data charting were conducted in accordance with PRISMA-ScR recommendations, ensuring transparent reporting of identification, screening, eligibility, and inclusion processes.

Data charting was performed using a standardized spreadsheet including metadata (author, year, country, journal), population and setting (undergraduate or service; discipline or unit; sample size), characteristics of the AI intervention (type, platform, duration or exposure, content and stages of the medication process covered, pedagogical components such as feedback and deliberate practice, need for human supervision, comparator when applicable), outcomes and measures (instruments, assessment times, metrics, scores, accuracy, time, errors, and, when reported, instrument validation), main results (direction or magnitude, statistical significance), and implementation aspects (acceptability, usability, adherence or engagement, barriers or facilitators). When multiple publications referred to the same intervention or sample, information was consolidated in the most comprehensive record.

In accordance with JBI guidance for scoping reviews, which emphasizes evidence mapping rather than critical appraisal of individual studies, no formal risk of bias assessment was performed. Instead, methodological limitations reported by the primary studies were described in the narrative synthesis. Data synthesis combined descriptive and tabular presentation (study characteristics, typology of interventions, outcomes or effects) with a structured narrative organized by type of intervention (for example, chatbots versus LLMs) and outcome domains (knowledge, performance, acceptability). This review was conducted and reported in accordance with the essential reporting items of the PRISMA-ScR checklist and the methodological guidance of the JBI.

## 3. Results

The database search across PubMed, Scopus, Web of Science, LILACS, and Google Scholar retrieved 545 records. After removing 136 duplicates, 409 unique records remained for screening. Of these, 389 were excluded based on title and abstract screening. Twenty full-text articles were assessed for eligibility, of which 18 were excluded (1 due to language and 17 due to irrelevant population or intervention), resulting in 2 studies included in the final synthesis.

The limited number of included studies reflects the novelty and narrow scope of research on the use of AI specifically for teaching safe medication administration in nursing. The search strategy was developed in accordance with PRISMA-ScR and JBI guidance and was reviewed by the research team to ensure sensitivity and appropriate use of Boolean operators. Previous scoping reviews in related areas have similarly reported a limited body of evidence, suggesting that this is an emerging field rather than a limitation of the search process.

**Figure 1:**
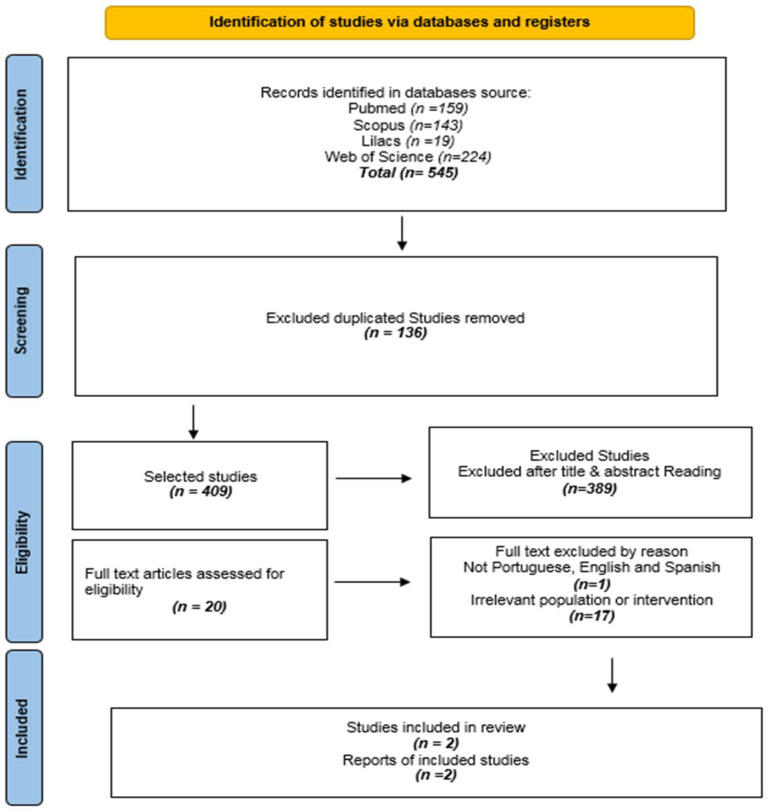
PRISMA-ScR flow diagram of study identification, screening, eligibility, and inclusion.

The PRISMA flow diagram depicts the review decision process (Figure 1).

The studies were conducted in Israel and South Korea, evaluated AI-based educational interventions targeting nursing students and professionals, and reported educational outcomes (knowledge, performance in tasks/simulations) and acceptability. No direct clinical outcomes were measured (for example, bedside medication errors or near misses). This absence of real-world safety indicators substantially limits the translation of the observed educational gains into evidence of improved clinical practice and highlights a critical gap in the current literature.

Table 1 organizes the contextual and methodological parameters, including country and year, setting (service or academia), sample size, design, target population, and thematic focus, that comprise the “where/who/how” axis. In both cases, these were single-center investigations, a condition that limits external validity and the generalizability of the findings.

Table 2 describes the design of the interventions. Chatbot-based solutions implement microlearning with deliberate practice and immediate feedback. LLM-based approaches function as dialogue-based tutoring, supporting the analysis of critical steps and the development of reasoning, contingent on human supervision and validation.

Table 3 summarizes the direction of effects, acceptability, and methodological robustness of the included studies, indicating improvements in knowledge and simulated performance, alongside important limitations related to external validity.

The included studies converge on consistent gains in knowledge and in performance on tasks or simulations related to the safe administration of medications, accompanied by favorable acceptability of AI-based solutions. In terms of pedagogical mechanisms, chatbot interventions operated in a microlearning format, emphasizing deliberate practice with immediate feedback; in turn, LLMs functioned as conversational tutors, enhancing reasoning and the review of critical steps in the medication process, provided they were used under human supervision to mitigate incomplete or ambiguous responses. Despite these positive signals, inferential strength remains limited by the absence of direct clinical outcomes (for example, errors or near misses), small samples and short intervention durations, as well as the scarcity of robust comparators and evaluations of curricular integration and impact in real clinical environments.

## 4. Discussion

To our knowledge, this scoping review is the first to synthesize applications of AI specifically aimed at teaching safe medication administration in nursing. The findings indicate that the use of AI in this domain remains nascent but promising. The identification of only two eligible studies underscores both the novelty of the field and the need for interpretive caution. Nevertheless, the observed effects, including cognitive gains and improved performance in simulated tasks related to dose calculation, checking, and preparation, are consistent with established evidence from the learning sciences and with recent findings on educational technologies in nursing [11,18].

In terms of measurable impact, the included studies reported quantitative improvements in educational outcomes, such as increased knowledge scores and enhanced performance in simulated medication administration tasks. The microlearning-based intervention demonstrated significant short-term gains in procedural accuracy in pre–post assessments, while the LLM-based study reported improvements in clinical reasoning and task completion in simulated scenarios. However, these outcomes were limited to educational and simulation-based measures, and no study evaluated direct effects on real-world medication error rates or near-miss events.

Within the instructional domain, microlearning-based interventions combined short modules, distributed practice, and immediate feedback, resulting in improvements in knowledge and procedural skills. This design aligns with evidence from reviews in health profession education reporting reduced cognitive load, improved retention, and better transfer to structured tasks [11]. Narrative syntheses in nursing education similarly indicate that brief, repetitive, and actionable modules enhance engagement and standardization of critical steps, particularly in high-risk areas such as medication administration [18]. The convergence with the broader literature lies in the pedagogical mechanism of task segmentation, deliberate practice, and immediate feedback. A key distinction, however, is that the interventions mapped in this review focused narrowly on procedural components of the medication process, whereas much of the existing literature addresses broader clinical content without explicitly linking learning outcomes to specific safety-critical steps.

In the generative domain, language models used as conversational tutors were associated with gains in clinical reasoning and structured verification of the “ten rights” in simulated contexts. Convergent literature characterizes LLMs as metacognitive mediators that externalize reasoning processes and prompt verification of assumptions, provided that faculty supervision ensures accuracy and adherence to institutional protocols [13]. Performance studies outside the educational setting, including comparisons between LLMs and professionals in pediatric dosage calculations, report high accuracy and shorter response times for well-defined tasks [13,19]. This helps explain the educational effect observed: when tasks are clearly structured, AI can offer effective scaffolding for decision-making. Importantly, while performance studies assess technical capability, the studies included here evaluated student learning, which depends on pedagogical integration and instructional design [13,19].

The integration of AI into academic education, particularly in generative applications, has the potential to reshape both teaching practices and routine cognitive tasks. However, anticipated gains in efficiency and responsiveness must be balanced against the need for data protection, user education, and systematic evaluation of unintended consequences associated with emerging technologies [15].

Regarding comparators, the included studies relied on pre–post designs without active pedagogical controls in the instructional domain or comparisons with human peers in the generative domain. This approach is consistent with the exploratory phase observed in related areas and is appropriate for proof of concept, yet it does not allow estimation of incremental benefits over established strategies such as structured simulation, problem-based learning, or non-AI checklists [19]. The literature recommends that future studies incorporate active comparators and randomization to strengthen causal inference and advance the field toward multicenter, longitudinal designs [19].

Acceptability and usability were consistently high. Conversational interfaces and brief microlearning modules appeared to reduce barriers, sustaining engagement and intention to use. Similar patterns are described in nursing education research, where concise and responsive content is associated with greater adherence and perceived usefulness [13,18]. This alignment may facilitate integration of such interventions into curricula and clinical placements, particularly when implementation costs are low.

The most significant gap identified, consistent with patient safety research, is the disconnect between proximal educational outcomes and objective clinical indicators. Evidence on medication safety emphasizes the importance of measuring concrete outcomes, such as error rates, near misses, and adherence to protocols, ideally in practice settings [19]. The studies mapped in this review remained at the cognitive and simulation level. This pattern reflects the developmental trajectory of educational research, which often begins with knowledge and Objective Structured Clinical Examination (OSCE) outcomes before progressing to chart audits or surveillance of adverse events. A critical next step, aligned with World Health Organization guidance on digital transformation and evidence-based learning, is to link AI-supported educational interventions to safety metrics in authentic clinical environments [5].

At the technopedagogical level, the findings align with ongoing discussions on explainability and governance of AI in healthcare. The educational value of LLMs increases when supported by curated reference sources, institutional protocols, and AI literacy among faculty and students, thereby reducing risks related to inaccurate outputs or bias [5,20]. The consistent presence of faculty supervision in the included studies reinforces this point: without structured oversight, tools designed to scaffold reasoning may inadvertently introduce errors and compromise safety.

Implications for nursing practice and education can be considered at three interconnected levels. At the procedural level, AI-supported repetition and feedback may reinforce standardized medication steps [11]. At the cognitive–strategic level, faculty-mediated use of generative tools may enhance reasoning and decision-making [13]. At the systems level, curricular integration, ongoing evaluation, and incorporation of safety indicators remain essential [5,19]. While the convergence with prior research supports the plausibility of the observed effects, the continued focus on procedural content, absence of clinical outcome measures, and lack of active comparators preclude claims of real-world error reduction and define a clear agenda for future research.

### 4.1. Practical Implications for Nursing Education and Curriculum Integration

The findings of this review have important practical implications for nursing educators seeking to integrate AI-based tools into curricula aimed at improving medication safety. Emerging evidence suggests that AI-supported educational environments can enhance personalization, promote self-regulated learning, and support the development of clinical reasoning when aligned with pedagogical objectives and institutional frameworks [8,14,19,21,22]. Recent studies indicate that structured integration of AI tools into nursing programs is associated with increased learner engagement, improved feedback mechanisms, and greater flexibility in skill acquisition, particularly in high-risk domains such as medication administration [21,23].

The current literature emphasizes that successful implementation requires intentional instructional design rather than ad hoc adoption of digital tools [22,24]. Studies published after the present search window highlight the importance of embedding AI-based interventions within competency-based curricula, combining automated feedback with faculty-mediated reflection and formative assessment [21,25]. This blended approach supports both procedural mastery and higher-order reasoning, reinforcing safe practices while preventing overreliance on automated systems [23,26].

From an operational perspective, educators are encouraged to prioritize pilot implementations within specific modules, such as pharmacology, fundamentals of nursing, and clinical skills laboratories, before scaling interventions across programs [24,27]. Evidence from recent evaluations demonstrates that phased implementation, supported by continuous monitoring and learner feedback, facilitates contextual adaptation and minimizes resistance to technological innovation [22,25]. Moreover, faculty development programs focused on AI literacy, ethical use, and instructional design have been identified as critical enablers of sustainable integration [21,26].

Institutional governance and data protection policies also play a central role in ensuring responsible adoption [23,28]. Contemporary studies underscore the need for transparent guidelines addressing data privacy, algorithmic bias, content verification, and accountability in AI-assisted learning environments [22,27]. Nursing educators must therefore collaborate with institutional leadership and information technology teams to establish regulatory frameworks that safeguard both learners and patients [24,28].

In addition, emerging research highlights the importance of aligning AI-based educational tools with assessment strategies and patient safety indicators [14,19,25]. Integrating simulation performance metrics, structured clinical examinations, and longitudinal competency evaluations can help bridge the gap between educational outcomes and clinical practice [21,23]. Such alignment supports the translation of knowledge gains into measurable improvements in professional performance and safety behaviors [8,26].

Taken together, these findings suggest that AI-based interventions should be implemented as components of coherent educational ecosystems rather than isolated technological solutions [22,24]. For nursing educators, this entails adopting evidence-informed instructional models, investing in faculty capacity building, and embedding continuous evaluation mechanisms [21,25]. When guided by pedagogical rigor and institutional support, AI tools can contribute meaningfully to the development of medication safety competencies and to the advancement of digitally enhanced nursing education [23,27,28].

### 4.2. Limitations

This scoping review has some limitations that should be acknowledged. First, although a comprehensive search strategy was applied across multiple databases and sources, the rapidly evolving nature of AI may have led to the omission of very recent studies or unpublished interventions. Second, the heterogeneity in study designs, educational contexts, AI technologies, and outcome measures limited comparability across studies and precluded deeper analytical synthesis. Third, most included studies relied on short-term educational outcomes and self-reported measures, which may overestimate perceived effectiveness. Thus, as a scoping review, this study did not assess methodological quality or risk of bias, and its findings should be interpreted as a mapping of available evidence rather than an evaluation of intervention effectiveness. Additionally, restricting the review to studies published in English, Portuguese, and Spanish may have led to the exclusion of relevant evidence from other linguistic contexts. Although this decision was necessary to ensure analytical rigor and feasibility, it may have introduced language bias and limited the global representativeness of the findings.

## 5. Conclusions

The mapping identified that the use of AI in teaching the safe administration of medications in nursing is an emerging field, with evidence concentrated in two complementary pedagogical approaches: instructional solutions to consolidate technical skills and generative solutions to support clinical reasoning and decision-making focused on patient safety. The available evidence suggests potential to strengthen competencies related to medication safety; however, important gaps remain regarding the demonstration of direct clinical impact in real-world contexts. Progressive and supervised adoption of these tools is therefore recommended, integrated into curricula and accompanied by continuous evaluation and structured initiatives to promote AI literacy, ethical awareness, and critical use among both faculty and students. To strengthen the scientific foundation of this field, future research priorities include multicenter studies with active pedagogical comparators, standardized outcome metrics, and the incorporation of objective patient safety indicators.

## Figures and Tables

**Table 1:** Characteristics and contextual features of the included studies on artificial intelligence in medication safety education.

Study	Country (Year)	Setting/Context	Population (N)	Design	Target Population	Central Theme
Study A	Israel (2024) [18]	Institutional educational program (education/service)	small sample (single center)	pre–post (feasibility)	newly hired nurses	safe medication administration
Study B	Public of Korea (2025) [19]	Nursing course/class (higher education)	n ≈ hundreds (single center)	cross-sectional (use evaluation)	nursing students (pediatric/neonatal)	safe medication administration supported by LLMs

Notes: N = sample size; LLMs = Large Language Models; pre–post = pre-intervention and post-intervention assessment.

**Table 2:** Characteristics and instructional components of artificial intelligence-based educational interventions for safe medication administration.

Study	Type of AI	Platform/Channel	Duration/Exposure	Comparator	Instructional Components
Study A [18]	Microlearning chatbot	App/educational messaging	Approximately 2 weeks (short, repeated sessions)	none (pre–post)	deliberate practice, immediate feedback, reinforcement of critical steps (calculation, checking, preparation/administration)
Study B [19]	Generative models (LLMs)	Conversational tool (ChatGPT/institutional LLM) ChatGPT https://chat.openai.com; accessed on 1 October 2025)	exposure during the course/activity	not described as a formal comparator	support for reasoning, review of safety steps, clarification of doubts; faculty supervision

Notes: AI = Artificial Intelligence; LLMs = Large Language Models; pre–post = pre-intervention and post-intervention assessment.

**Table 3:** Educational outcomes, acceptability, and methodological limitations of the included studies.

Study	Educational Outcomes	Observed Effect	Acceptability/Usability	Patient Safety Measures	Main Limitations
Study A [18]	Knowledge; performance in simulations/tasks (calculation, checking, preparation/administration)	improved knowledge and performance post-intervention	high (short-term adherence; positive feedback)	not measured (only indirect indications via simulated performance)	small sample; single center; no comparator; short intervention
Study B [19]	Use/experience with LLMs; self-perceived learning	positive signal for learning support; need for curation	good (perceived usefulness; ease of use)	not measured (no clinical data)	single sample; self-report; lack of evaluation in real clinical settings

Notes: LLMs = Large Language Models.

## Data Availability

Data sharing is not applicable. No new data were created or analyzed in this study.

## Public Involvement Statement

No public involvement was associated with this research.

## Guidelines and Standards Statement

This manuscript was conducted and reported in accordance with the Preferred Reporting Items for Systematic Reviews and Meta-Analyses extension for Scoping Reviews (PRISMA-ScR).

## Abbreviations

The following abbreviations are used in this manuscript:

AI	Artificial Intelligence
LLMs	Large Language Models
JBI	Joanna Briggs Institute
PRISMA-ScR	Preferred Reporting Items for Systematic Reviews and Meta-Analyses Extension for Scoping Reviews
WHO	World Health Organization
DeCS	Health Sciences Descriptors
MeSH	Medical Subject Headings
OSF	Open Science Framework
OSCEs	Objective Structured Clinical Examinations
T&L	Teaching and Learning

## Appendix A

**Table A1:** Search strategies used in the scoping review.

Database	Search Strategy
PubMed/MEDLINE	(“Artificial Intelligence”[MeSH] OR “machine learning” OR “deep learning” OR chatbot* OR “large language model*” OR “generative AI”) AND (“Medication Errors”[MeSH] OR “Medication Administration”[MeSH] OR “medication safety” OR “drug administration”) AND (“Nursing”[MeSH] OR “nursing education” OR nurses). Filters: publication year ≥ 2010; languages English, Portuguese, and Spanish.
Scopus	TITLE-ABS-KEY (“artificial intelligence” OR “machine learning” OR “deep learning” OR chatbot* OR “large language model*” OR “generative AI”) AND TITLE-ABS-KEY (“medication administration” OR “medication safety” OR “medication error*”) AND TITLE-ABS-KEY (nursing OR “nursing education”).
Web of Science Core Collection	TS = (“artificial intelligence” OR “machine learning” OR chatbot* OR “large language model*” OR “generative AI”) AND TS = (“medication administration” OR “medication safety” OR “medication error*”) AND TS = (nursing OR “nursing education”).
LILACS	(“inteligência artificial” OR “artificial intelligence” OR chatbot* OR “modelo de linguagem”) AND (“administração de medicamentos” OR “erros de medicação” OR “segurança do paciente”) AND (enfermagem).
Google Scholar	“artificial intelligence” AND nursing AND (“medication administration” OR “medication safety”). The first pages of results sorted by relevance were screened.

Notes: The asterisk (*) indicates truncation used to retrieve variations of the word.
